# The impact of working as a peer worker in mental health services: a longitudinal mixed methods study

**DOI:** 10.1186/s12888-022-03999-9

**Published:** 2022-06-01

**Authors:** Steve Gillard, Rhiannon Foster, Sarah White, Sally Barlow, Rahul Bhattacharya, Paul Binfield, Rachel Eborall, Alison Faulkner, Sarah Gibson, Lucy P. Goldsmith, Alan Simpson, Mike Lucock, Jacqui Marks, Rosaleen Morshead, Shalini Patel, Stefan Priebe, Julie Repper, Miles Rinaldi, Michael Ussher, Jessica Worner

**Affiliations:** 1 City, University of London, London, UK; 2 St George’s, University of London, London, UK; 3 East London NHS Foundation Trust, London, UK; 4 South London & Maudsley NHS Foundation Trust, London, UK; 5 Independent Survivor Researcher, London, UK; 6 King’s College London, London, UK; 7 University of Huddersfield, Huddersfield, UK; 8 South West London & St George’s Mental Health NHS Trust, London, UK; 9 Queen Mary, University of London, London, UK; 10 Implementing Recovery through Organisational Change, Nottingham, UK; 11 University of Stirling, Stirling, UK; 12 National Suicide Prevention Alliance, London, UK

**Keywords:** Peer support, Mental health services, Wellbeing, Employment, Job satisfaction, Burnout, Interdisciplinary team working, Mixed methods research, Psychiatric inpatient care, Community mental health

## Abstract

**Background:**

Peer workers are increasingly employed in mental health services to use their own experiences of mental distress in supporting others with similar experiences. While evidence is emerging of the benefits of peer support for people using services, the impact on peer workers is less clear. There is a lack of research that takes a longitudinal approach to exploring impact on both employment outcomes for peer workers, and their experiences of working in the peer worker role.

**Methods:**

In a longitudinal mixed methods study, 32 peer workers providing peer support for discharge from inpatient to community mental health care - as part of a randomised controlled trial - undertook in-depth qualitative interviews conducted by service user researchers, and completed measures of wellbeing, burnout, job satisfaction and multi-disciplinary team working after completing training, and four and 12 months into the role. Questionnaire data were summarised and compared to outcomes for relevant population norms, and changes in outcomes were analysed using paired t-tests. Thematic analysis and interpretive workshops involving service user researchers were used to analysis interview transcripts. A critical interpretive synthesis approach was used to synthesise analyses of both datasets.

**Results:**

For the duration of the study, all questionnaire outcomes were comparable with population norms for health professionals or for the general population. There were small-to-medium decreases in wellbeing and aspects of job satisfaction, and increase in burnout after 4 months, but these changes were largely not maintained at 12 months. Peer workers felt valued, empowered and connected in the role, but could find it challenging to adjust to the demands of the job after initial optimism. Supervision and being part of a standalone peer worker team was supportive, although communication with clinical teams could be improved.

**Conclusions:**

Peer workers seem no more likely to experience negative impacts of working than other healthcare professionals but should be well supported as they settle into post, provided with in-work training and support around job insecurity. Research is needed to optimise working arrangements for peer workers alongside clinical teams.

## Introduction

Peer support is what many people do when they recognise a shared experience of adversity and choose to support each other [1]. While people might share many things in common, relating to experiences, interests, personal identity or to community [2], some shared experience of mental distress or of using mental health services is core to mental health peer support [3]. Within mental health services, peer support is increasingly provided by people trained and paid – as peer workers – to make use of their own experiences of mental distress in supporting others with similar experiences as part of their mental health care. Peer workers are employed in a range of roles: paraclinical roles such as healthcare assistants or community support workers; providing structured support for self-management; or in a more loosely structured capacity as part of a more personal recovery process [4]. There is increasing evidence of the benefits of one-to-one peer support in mental health services, with a recent systematic review and meta-analysis of data from 19 trials of one-to-one peer support indicating a significant improvement in self-reported recovery and sense of empowerment for people offered peer support compared with care as usual [5].

A number of studies also point to benefits for peer workers, while at the same time stressing caution about potential negative impacts. An early scoping review of the literature on peer support in mental health services suggested that peer support might enhance personal recovery for peer workers, but could also be a source of stress, with mental health professionals voicing concern that the peer workers they work alongside might relapse and be hospitalised because of the stresses of the role [6]. A review of qualitative research about peer workers’ experiences of peer support indicated improvements in confidence, self-esteem and social contacts for peer workers [7]. A qualitative interview study with 31 peer workers, working in a variety of mental health services, suggested that benefits included improvements in mental illness management and general health, emotional and spiritual wellness, enhanced interpersonal relations, vocational recovery and professional development [8]. A review of qualitative research focused on the impact of peer support on peer workers indicates that peer workers experience enhanced personal recovery relating to improved knowledge about their mental health and a new positive sense of self relating to the role, but these improvements can be undermined where acceptance, value and support for the role is absent within the clinical team [9].

A wider literature explores the impact of implementation issues and organisational environment on peer support in mental health services [10], suggesting that the potential benefits – for people offered peer support - can become diluted where key aspects of how peer support is put into practice are poorly defined [11]. Notably, it has been identified that a clear peer worker role description [12], role specific training and support [13, 14], preparation for clinical teams working alongside peer workers [15], and shared expectations of the peer worker role across peer workers and their clinical colleagues [11] all facilitate successful delivery of peer support. Poor quality implementation, in particular in relation to the role of the peer worker as part of the multi-disciplinary clinical team, has also been shown to impact outcomes for peer workers. A qualitative interview study based in an inpatient setting in Germany showed that peer workers experienced pressure to succeed as pioneers in a new role, had to negotiate identity issues with existing professional staff - as colleague, rival or patient – and had to navigate unfamiliar issues around information sharing, boundaries and professionalism [16]. In Canada, Voronka [17], an experienced peer worker and researcher, writes of the demands on peer workers of having to perform a marginalised, experiential identity while at the same time following professional rules and regulations – to pass simultaneously as both normal and disabled – echoing research from the UK [18]. A qualitative interview study with 23 peer workers and 11 ‘non-peer’ mental health workers in the US suggests that levels of job satisfaction among peer workers are contingent on role clarity, a sense of autonomy in the role and acceptance by non-peer co-workers [19].

While much of this research is qualitative and focuses on an experiential perspective, efforts have been made to quantify the impacts of working as a peer worker. A survey of 84 peer workers working in a range of mental health services in one state in the US found that peer workers experienced difficulties including poor financial compensation, limited employment opportunities, work stress, the emotional stress of helping others and in maintaining personal wellness, with 44% reporting having a relapse in their mental health while working as a peer worker [20]. Another survey of 253 peer workers in the US indicated benefits of working in the role as increased self-efficacy and self-esteem, enhanced recovery, better communication and sense of belonging, as well as a new sense of meaning in life, with negative impacts related to poor pay and workplace opportunities [21]. Development of a scale to measure job satisfaction for peer workers indicates intangible and tangible factors of satisfaction, with intangible benefits including relational aspects of work, sense of self-efficacy and accessible work environment, and tangible benefits comprising payment, promotion and educational opportunities [22]. A survey of 597 peer workers across the US found that over half were very satisfied with their work (with another third somewhat satisfied), and that feeling respected, being given responsibility, having sufficient training and support, and opportunity to use skills were all significant predictors of satisfaction [23].

Burnout in the workplace - characterised by emotional exhaustion, depersonalisation and a diminished sense of personal accomplishment [24] – has been identified as an issue effecting a higher proportion of mental health workers than other healthcare professionals [25], with the implications of burnout including increased staff turnover and absences as well as poorer job performance [26]. A small number of studies have compared burnout and other employment-related outcomes for peer workers with those of other mental health workers. A longitudinal study of burnout among 152 peer workers in veterans’ mental health in the US indicated that levels of burnout were similar to other mental health workers and that, while levels of burnout increased in the first 6 months of employment, this increase was not observed over the first year of employment [27]. A recent survey of 67 peer workers in one state in Australia indicated that job satisfaction, burnout and turnover intention for peer workers was not significantly different to other mental health workers [28]. In contrast, recent benchmarking data from the UK, among 862 peer workers in National Health Service (NHS) mental health services, indicated substantially higher levels of staff absence and turnover than in the NHS workforce as a whole [29]. While the assumption that peer workers are inherently vulnerable in the work place and will inevitably spend more time on sick leave than other workers has been challenged as a myth [30], this range of findings suggests that differential working environment might be associated with outcomes for peer workers.

We note a growing body of evidence describing the impact of peer working on a range of outcomes for peer workers, with many studies considering employment-related outcomes as well as outcomes relating to mental health, personal recovery and other psychosocial outcomes. While much of this research is qualitative, more recent quantitative studies begin to explore the relationship between employment outcomes and working conditions for peer workers. However, these studies are almost exclusively cross-sectional and there are no studies that combine quantitative and qualitative enquiry in order to understand how the way in which peer support is implemented into practice in mental health services impacts on employment-related outcomes for peer workers. This study aims to address that knowledge gap and so contribute to research informing best practice in supporting peer workers in their role. The paper addresses the follow research questions:

1. What is the impact of working as a peer worker on employment-related outcomes and wellbeing, and how does this change over time?
2. How do peer workers experience the impact of peer working on their work and wellbeing, and how does this change over time?

## Method

This is a mixed method, longitudinal study using standardised measures of outcome, structured questionnaires, and semi-structured qualitative interviews. All methods were carried out in accordance with relevant guidelines and regulations. The study was informed by experiential knowledge of mental health and peer support [31], as well as clinical and academic knowledge, with service user researchers on the research team - many of whom had a range of experiences of giving and receiving peer support - playing a key role in developing interview questions, conducting interviews and analysing data.

### Setting

The study took place in seven NHS mental health services in England. Services were study sites in a large randomised controlled trial of peer support for discharge from inpatient to community mental health care (trial registration: 10.1186/ISRCTN10043328). Peer support was provided by peer workers, recruited and trained specifically to provide peer support for discharge. A detailed description of the peer worker role, training provided and the support and supervision they received in post is given in the study protocol [32]. In brief, people admitted to psychiatric inpatient care were offered at least one meeting with a peer worker while in hospital, prior to discharge, and then weekly meetings for 10 weeks in the community following discharge and a further three fortnightly meetings. Peer support principles [33] underpinned a peer support handbook and training programme, the development of which was coproduced by peer workers, service user researchers and a Lived Experience Advisory Panel alongside other academics on the research team, and clinical staff in study sites [34]. The training provided guidance and practice for peer workers on building a relationship based on shared experiences, using their experiential knowledge in the role, a range of ‘strength-based’ tools that they could use flexibly with the people they supported, and enabling people to make and strengthen connection with community resources. Peer workers were supervised and supported by a peer worker coordinator (PWC), an experienced peer worker with team leadership expertise. Peer workers were based in a dedicated peer support team at each site, working across inpatient and community mental health services, with approximately two full-time equivalent roles shared between two to five part-time peer workers per site. A full-time equivalent peer worker provided support to up to eight people through the discharge process at any one time, with face-to-face contacts lasting for up to 2 h supplemented with telephone contacts as necessary. Other duties included a weekly team meeting, administrative tasks associated with the role, any ongoing training provided by the service provider, and meeting with clinicians as agreed with the people they were supporting.

### Sample and recruitment strategy

All 32 peer workers who provided peer support in the trial were given information about the study and invited to give written, informed consent to take part by a member of the study team.

### Data collection

Interviews took place at three time points: after training and induction into the role (T1); after 4 months in post (T2); after 12 months in post (T3). At T1 only, the interview included structured questions collecting socio-demographic data. At T1 and T3 structured questions were asked about each participant’s use of mental and physical healthcare services for the preceding 3 months using a modified version of the Client Services Receipt Inventory (CSRI) [35]. At all three time-points participants were asked to complete the following standardised measures:

1. Warwick-Edinburgh Mental Wellbeing Scale (WEMWBS) [36] – a well validated, self-report scale designed to measure positive mental health;
2. Job Satisfaction Measure [37] – a self-report scale developed for community nurses, including subscales of personal satisfaction, workload, professional support, salary and training;
3. Interdisciplinary Team Process and Performance in Long-term Care [38] – a scale assessing experience of healthcare team working including domains of leadership, communication, coordination, conflict management, team cohesion and perceived team effectiveness, demonstrating a high degree of reliability and validity across care settings;
4. Maslach Burnout Inventory [39] – a well-validated and reliable self-report scale used in mental health services [40], demonstrating inverse correlation with job satisfaction, and positive correlation with absenteeism, lower productivity and staff turnover [24].

Data on days absent from work and length of employment on the peer support for discharge project for each peer worker was collected from the PWC at each site. Data on number, length and type (face-to-face or telephone) of contact with each person supported by each peer worker was collected from an online contact log completed by peer workers after each contact.

Peer workers completed a qualitative interview, lasting about an hour, with a service user researcher on the study team at each timepoint (see supplementary material). Interviews at T1 explored peer workers’ experiences of recruitment to the role and training received, how well they thought training and induction had prepared them for the role, and initial experiences of being part of a peer worker team. Interviews at T2 and T3 explored peer workers’ experiences of providing peer support, reflection on how training helped them prepare for the role, challenges and rewards of the role, experiences of team working and working alongside clinical teams, experiences of support and supervision, and reflections on staying well at work. The interview at T3 also considered future work aspirations and opportunities. Qualitative interview schedules were developed using the available literature (cited above) and the expertise of the study team, including the experiential knowledge of service user researchers and team members involved in developing and delivering peer support. Input from the study Lived Experience Advisory Panel and peer workers involved in the pilot trial [32, 34] also helped inform the development of research questions.

### Ethical approval

Approval was granted by the UK National Research Ethics Service, Research Ethics Committee London - London Bridge on 10 May 2016, reference number 16/LO/0470. Written, informed consent was given by all research participants.

### Quantitative data analysis

The socio-demographic characteristics of the peer worker sample are described, with frequencies and percentages for categorical variables, and mean and standard deviation (SD), and minimum and maximum values for continuous variables. Count data only are provided for service use given low numbers of participants. Outcome measures are summarised by mean and standard deviation (SD), minimum and maximum values, and reported alongside representative normative data. Normative data were chosen to reflect either the general population (wellbeing) [41] or a large sample (greater than 500) of healthcare professionals from the UK (job satisfaction [42] and burnout [43]) or USA (interdisciplinary team working [38]). Comparison of the outcomes for peer workers to normative samples was by inspection rather than tested statistically. To examine change in outcomes over a year, paired t-test analyses were conducted comparing T1 to T3 data and T1 to T2 data. Results are reported by mean differences (change) and 95% confidence intervals (CI), the *p*-value and the effect size (ES) (calculated by the mean difference divided by the T1 SD for the respective measure). If a subscale of a measure had more than one item missing it was set to missing. All statistical analysis was conducted in IBM SPSS Statistics v26.

### Qualitative data analysis

Qualitative data analysis proceeded in stages, first generating a largely descriptive coding framework that organised and made sense of peer workers experiences of the role, and second, a more explicitly explanatory analysis that sought meaning in those experiences [44]. Given that peer support is predicated on the use and sharing of experiential knowledge of mental distress [45], we sought to integrate the experiential perspective brought by service user researchers in the team throughout the process, alongside the clinical and academic perspectives of other team members [46].

In the first stage, members of the research team each undertook a preliminary coding of one or two interview transcripts. We then held an interpretive workshop, with each team member sharing and explained emerging analytical ideas from their preliminary coding. Analytical ideas were explored by the team, with ideas integrated as potential codes where they were meaningfully similar (idiosyncratic ideas remained as separate codes) to produce a provisional coding framework. A second round of preliminary coding of new transcripts combined deductive and inductive approaches [47]. The provisional framework was applied, deductively, to transcripts to explore the fit between data and codes, with transcripts also analysed inductively to allow new analytical ideas to emerge. In a second interpretive workshop, team members again presented their preliminary analyses and, through discussion as a team, existing codes were modified where necessary and new codes added to refine the coding framework. Service user researchers on the team then used the refined framework to code the full set of peer worker interviews using NVivo qualitative analysis software.

In the second stage, two members of the team (RF and SG) first wrote interpretive memos around the data within each code of the framework before, through rounds of discussion, exploring and identifying a smaller number of themes that offered explanation [44] of how and why peer workers were impacted by their work. Emerging themes were shared with the full team and refined through further discussion.

### Data synthesis

We adopted a Critical Interpretive Synthesis approach to data synthesis, as has been widely used to synthesise quantitative and qualitative evidence in systematic reviews [48] and the development of evidence-based practice [49, 50]. In this approach findings of different analyses (i.e. descriptive analysis and hypothesis testing of quantitative data, and thematic analysis of qualitative data) are mapped onto a grid that explores how those analyses interface. This enables the development of synthesising arguments or propositions that offer explanatory insight into study findings as a whole and inform applied learning from the research. Synthesis was initially undertaken by SG and RF and then refined through discussion with the full team.

## Results

### Sample characteristics

Thirty-one peer workers employed in the ENRICH trial competed a baseline interview. One further peer worker completed data at T2 only so their socio-demographic and outcome data has been used at T1. Participant characteristics are reported in Table 1.

**Table 1:** Demographic characteristics

		n	n (%)
**Site**	**S1**	32	5 (15.6%)
	**S2**		7 (21.9%)
	**S3**		6 (18.8%)
	**S4**		5 (15.6%)
	**S5**		3 (9.4%)
	**S6**		3 (9.4%)
	**S7**		3 (9.4%)
**Gender**	**Male**	32	10 (31.3%)
	**Female**		21 (65.6%)
	**Prefer not to say**		1 (3.1%)
**Sexual orientation**	**Bisexual**	31	3 (9.7%)
	**Lesbian/Gay**		2 (6.5%)
	**Heterosexual**		22 (71.0%)
	**Prefer not to say**		4 (12.9%)
**Ethnicity**	**White British**	30	20 (66.7%)
	**White Irish**		3 (9.0%)
	**White other**		2 (6.7%)
	**Arab**		1 (3.1%)
	**Black/Black British African**		1 (3.1%)
	**Asian/Asian British Pakistani**		1 (3.1%)
	**Mixed White & Asian**		1 (3.1%)
	**Mixed other Mixed background**		1 (3.1%)
**Highest education level**	**Secondary school**	25	7 (28.0%)
	**More than secondary school**		10 (40.0%)
	**University graduate**		4 (16.0%)
	**Post-graduate**		4 (16.0%)
**Marital status**	**Married**	25	3 (12.0%)
	**Single**		16 (64.0%)
	**Divorced**		2 (8.0%)
	**In a relationship**		4 (16.0%)
**Religion**	**No religion**	28	14 (50.0%)
	**Christian**		8 (28.6%)
	**Muslim**		3 (10.7%)
	**Religion not stated**		3 (10.7%)
**English is first language**		25	24 (96.0%)
**Has child under 18 years living in household**		23	1 (4.3%)
			**Mean (SD) Min-Max**
**Age**		27	42.9 (9.0) 26.0–59.0
**Length of time in post, months**		32	17.7 (8.2) 6.1–31.6
**Number of contracted hours per week**		30	17.8 (5.6) 10.0–30.0
**Number of days sick leave**		30	7.7 (13.8) 0.0–55.0
**Number of peers**		30	9.7 (8.0) 1.0–40.0
**Number of completed face to face contacts**		30	56.1 (54.0) 2.0–273.0

Key: SD = standard deviation; S = site

Peer workers comprised 21 (66%) women and had a mean age of 42.9 (standard deviation 9.0) ranging from 26 to 59. Twenty-five out of the 30 peer workers (83%) who reported their ethnicity were of white ethnicity. Eight out of 25 peer workers (32%) were graduates and seven out of 25 (28%) were married or in a relationship.

The 32 peer workers were in post for 17.7 months on average, ranging from 6.1 to 31.6 months. Two of the peer workers were employed as bank (causal) staff so had no contracted hours or recorded sick leave. For the 30 peer workers who were contracted the mean number of hours worked per week was 17.8 hours, ranging from 10 to 30 hours per week. Twelve of the thirty peer workers (38%) had no recorded sick leave, with mean sick leave for all peer workers of 7.7 days. The remaining 18 peer workers had a median number of sick days of five, ranging from 0 to 55 days.

Two of the 32 peer workers did not record their contacts with peers. The 30 peers with recorded contacts were assigned a mean of 9.7 people to support, ranging from one to 39 people supported by individual peer workers. There was a wide range in the number of completed face-to-face contacts for each peer worker, from two to 273, with a mean of 56.1 contacts. Peer workers conducted 1682 completed face-to-face contacts over the course of the study.

Use of mental and physical health services 3 months prior to T1 was characterised by routine care, with no peer workers reporting an inpatient psychiatric admission or use of crisis or emergency care for their mental health (see Table 2).

**Table 2:** Peer worker mental and physical healthcare service use

	T1 (***n*** = 32) No. of peer workers reporting contact	T3 (***n*** = 21) No. of peer workers reporting contact
Hospital admission (mental health)	0	0
Hospital admission (physical health)	1	0
A&E attendance (mental health)	0	0
A&E attendance (physical health)	4	0
Outpatient visit (mental health)	5	0
Psychiatrist	7	3
Community mental health services	13	6
Crisis & home treatment team	0	0
Psychological therapy	4	7
GP	13	10
Primary care nurse	8	3
Wellbeing services (community-based)	9	12

Key: T1 = timepoint 1 (post-training); T3 = timepoint 3 (12 months post-training); *A&E *accident and emergency department; *GP *general practitioner

### Quantitative analysis

#### Measures of outcome

Data were available for 20 peer workers at T2 and 21 at T3. Table 3 reports descriptive statistics of all outcome measures at each time point. Wellbeing of peer workers remained fairly constant over the year, marginally lower than the general population norm. Job satisfaction subscales and overall score mean values appeared higher than the norm sample apart from satisfaction with pay and prospects subscales. Interdisciplinary Team Scale subscales were higher than the norm values across the three timepoints apart from team effectiveness and workplace resources. Mean scores on the Maslach Burnout Inventory subscales indicated lower levels of burnout and depersonalization, and slightly higher levels of personal effectiveness than norms.

**Table 3:** Summary statistics of outcomes over time and compared with norm data

		T1		T2		T3		
		n	Mean (SD)	n	Mean (SD)	n	Mean (SD)	Norm
**Wellbeing**^a^		32	49.8 (9.07) 28.0–70.0	20	47.7 (9017) 32.0–69.0	21	48.7 (11.73) 25.0–68.0	51.6 (8.71)
**Measure of Job Satisfaction**^b^	**Personal Satisfaction**	29	4.3 (0.72) 1.5–5.0	17	4.2 (0.51) 3.3–5.0	19	4.2 (0.64) 3.2–5.0	3.7 (0.65)
	**Satisfaction with Workload**	30	3.9 (0.76) 1.8–5.0	17	3.8 (0.62) 2.5–4.9	19	3.8 (0.78) 2.6–5.0	3.1 (0.77)
	**Satisfaction with** **Professional Support**	31	4.5 (0.53) 3.0–5.0	17	4.5 (0.58) 3.4–5.0	19	4.4 (0.82) 1.8–5.0	3.6 (0.69)
	**Satisfaction with Training**	29	3.9 (0.74) 1.6–5.0	17	3.8 (0.92) 2.4–5.0	19	3.6 (0.86) 2.2–5.0	3.2 (0.92)
	**Satisfaction with Pay**	30	3.5 (1.14) 1.0–5.0	17	3.4 (1.23) 1.0–5.0	20	3.5 (1.10) 1.0–5.0	3.4 (0.66)
	**Satisfaction with Prospects**	30	3.5 (0.92) 1.2–5.0	17	3.5 (1.00) 1.7–5.0	19	3.1 (1.10) 1.0–4.8	3.4 (0.66)
	**Satisfaction with** **Standards of Care**^**d**^	29	4.0 (0.62) 2.8–5.0	17	4.0 (0.65) 2.3–5.0	19	3.9 (0.89) 2.2–5.0	
	**Overall Satisfaction**	30	3.9 (0.66) 1.9–5.0	17	3.9 (0.60) 3.0–4.7	20	3.7 (0.74) 2.6–5.0	3.44 (0.53)
**Inter-disciplinary Team Survey**^c^	**Leadership**	31	4.2 (0.66) 2.2–5.0	20	4.4 (0.47) 3.3–4.9	21	4.1 (0.62) 2.7–5.0	3.8 (0.77)
	**Team Cohesion**	32	4.3 (0.84) 1.6–5.0	20	4.3 (0.47) 3.3–5.0	21	4.2 (0.93) 1.7–5.0	4.0 (0.73)
	**Communication**	31	4.1 (0.57) 2.5–5.0	20	4.0 (0.60) 2.8–5.0	21	3.8 (0.93) 1.5–5.0	3.6 (0.69)
	**Coordination**	31	4.2 (0.66) 1.8–5.0	20	4.2 (0.61) 2.8–5.0	21	4.1 (0.86) 1.7–5.0	3.9 (0.75)
	**Conflict Management**	27	3.8 (0.49) 2.8–4.7	20	3.8 (0.35) 3.2–4.4	20	3.7 (0.45) 2.7–4.3	3.6 (0.66)
	**Team Effectiveness**	26	4.0 (0.36) 3.0–4.4	20	3.9 (0.40) 3.0–4.4	19	3.7 (0.59) 2.3–4.4	4.2 (0.69)
	**Workplace Conditions**	31	3.7 (0.83) 1.4–5.0	19	3.9 (0.73) 2.2–5.0	20	3.8 (0.80) 2.4–5.0	3.2 (0.91)
	**Workplace Resources**	27	3.6 (1.01) 1.6–5.0	18	3.6 (0.89) 1.6–5.0	18	3.5 (0.74) 2.2–5.0	3.8 (0.86)
**Maslach Burnout Inventory**^d^	**Emotional exhaustion**	32	8.6(9.27) 0.0–39.0	20	9.8 (7.84) 0.0–28.0	21	11.8 (9.72) 1.0–32.0	19.7 (9.6)
	**Depersonalization**	32	3.0 (3.61) 0.0–14.0	20	4.4 (3.90) 0.0–16.0	21	4.7 (4.07) 0.0–14.0	8.9 (7.4)
	**Personal Achievement**	23	39.3 (7.34) 18.0–48.0	19	38.7 (6.90) 24.0–48.0	19	37.5 (11.2) 17.0–56.0	35.8 (7.6)

Key: T1 = timepoint 1 (post-training); T2 = timepoint 2 (4 months post-training); T3 = timepoint 3 (12 months post-training); ^a^Wellbeing norm sample taken from 2011 Health Survey for England (*n* = 7020) [41]; ^b^ Satisfaction with Standards of Care subscale was missing from the version used in the paper from which we have taken population norms (*n* = 534) [42]; ^c^ Interdisciplinary team survey norm sample are part-time and full-time employees who had direct patient care responsibilities in US long term care facilities for the elderly (*n* = 1152)38; ^d^Burnout norm data taken from a UK sample of nurses (*n* = 9855) [43]

#### Change in service use

While it was not possible to estimate the effect of working as a peer worker on mental and physical healthcare use because of low numbers of participants, a similar pattern was observed in the 3 months to T3, compared to the 3 months to T1 with continued emphasis on routine rather than acute or emergency mental health care (Table 2).

#### Change in outcomes

Change in outcomes from T1 to T2, and T1 to T3 is reported in Table 4. Between baseline and 4 months follow-up there was a statistically significant decrease of nearly 4 points in wellbeing, a medium effect size, 0.56. However, over the course of the year there was no significant change in wellbeing.

**Table 4:** Change in outcomes over time

		T1 – T2				T1 – T3			
		n	Change (95% CI)	*p*-value	ES	n	Change (95% CI)	*p*-value	ES
**Wellbeing**		20	3.9 (0.67, 7.13)	0.020	0.56	21	1.00 (−3.46, 5.46)	0.645	0.09
**Measure of Job Satisfaction**	**Personal Satisfaction**	16	0.2 (0.00, 0.44)	0.044	0.50	19	0.09 (− 0.28, 0.46)	0.625	0.11
	**Satisfaction with Workload**	16	0.2 (0.06, 0.39)	0.012	0.31	19	0.14 (−0.12, 0.40)	0.283	0.17
	**Satisfaction with Professional Support**	17	0.1 (− 0.03, 0.32)	0.099	0.24	20	0.19 (− 0.15, 0.53)	0.254	0.33
	**Satisfaction with Training**	17	0.2 (−0.11, 0.58)	0.168	0.32	20	0.39 (0.03, 0.76)	0.036	0.46
	**Satisfaction with Pay**	16	0.0 (−0.28, 0.33)	0.859	0.00	19	0.04 (−0.32, 0.40)	0.820	0.04
	**Satisfaction with Prospects**	16	0.3 (0.09, 0.42)	0.004	0.33	19	0.45 (0.01, 0.89)	0.047	0.43
	**Satisfaction with** **Standards of Care**	16	0.0 (−0.30, 0.28)	0.940	0.00	19	0.13 (−0.21, 0.48)	0.434	0.22
	**Overall Satisfaction**	17	0.1 (−0.01, 0.29)	0.069	0.19	20	0.23 (−0.04, 0.50)	0.096	0.31
**Inter-disciplinary Team Survey**	**Leadership**	20	0.0 (−0.30, 0.25)	0.857	0.00	21	0.07 (−0.26, 0.40)	0.677	0.12
	**Team Cohesion**	20	0.1 (−0.35, 0.47)	0.757	0.14	21	0.04 (−0.51, 0.60)	0.880	0.05
	**Communication**	20	0.2 (−0.05, 1.60)	0.131	0.38	21	0.37 (−0.03, 0.76)	0.069	0.86
	**Coordination**	20	0.1 (−0.18, 0.41)	0.417	0.18	21	0.20 (−0.25, 0.66)	0.363	0.36
	**Conflict Management**	20	0.1 (−0.17, 0.28)	0.616	0.17	21	0.13 (−0.09, 0.36)	0.235	0.22
	**Team Effectiveness**	19	0.1 (−0.04, 0.34)	0.122	0.32	20	0.20 (−0.11, 0.50)	0.191	0.48
	**Workplace Conditions**	18	0.0 (−0.40, 0.38)	0.952	0.00	20	−0.01 (− 0.49, 0.47)	0.965	−0.01
	**Workplace Resources**	18	0.1 (−0.57, 0.69)	0.840	0.11	20	0.00 (−0.38, 0.38)	0.982	0.00
**Maslach Burnout Inventory**	**Emotional exhaustion**	20	−2.0 (−4.00, 0.02)	0.052	−0.27	21	−2.71 (−5.89, 0.46)	0.090	− 0.26
	**Depersonalization**	20	−1.5 (− 2.60, − 0.42)	0.009	− 0.48	21	−1.29 (− 2.51, − 0.06)	0.040	−0.32
	**Personal Achievement**	14	1.7 (−1.00, 4.41)	0.192	0.27	13	3.23 (−0.94, 7.40)	0.117	0.48

Key: T1 = timepoint 1 (post-training); T2 = timepoint 2 (4 months post-training); T3 = timepoint 3 (12 months post-training); *CI *confidence interval; *ES *effect size

With respect to job satisfaction there was a statistically significant decrease in the following subscales at the 4-month follow-up; personal satisfaction (medium effect size, 0.50), satisfaction with workload (small effect size, 0.31) and satisfaction with prospects (small effect size, 0.33). This decrease in satisfaction with prospects continued over the year with T1 to T3 scores reducing by 0.45 points, a statistically significant change (small - medium effect size, 0.43). Over the course of the year there was also a statistically significant decrease in satisfaction with training (small - medium effect size, 0.46).

There was no statistically significant change in any of the Interdisciplinary Team subscales at four-month follow-up or 1 year. Of note however is the relatively large effect size for a drop in the Communication subscale score, 0.38 to T2 and 0.86 to T3.

Examining the Maslach Burnout Inventory, there was a significant increase in depersonalization at both T2 and T3, the increase occurring in the first 4 months and seemingly maintained to T3 (small-medium effect size to T2, − 0.48).

### Qualitative analysis

Our descriptive analysis produced a coding framework with 18 codes from which we derived three explanatory-level themes reflecting positive, negative and complex experiences of impact. Our thematic analysis is presented below with illustrative data from interview transcripts. Participants are identified with an identifier comprising site number (e.g. S1 = site 1), participant number for that site (e.g. PW01 = peer worker 1) and timepoint for the interview (e.g. T1 = timepoint 1).

#### Feeling valued

Peer workers described a sense of feeling valued as an important impact of taking on the role, of finding meaning, as an individual, through using their experiences of mental health and what they offered as a peer worker in supporting others:

For me it was about having the opportunity to be able to help others and to be able to realise that I was a human being who was valued … So then when I started realising that I am a valuable person and I’ve got skills and I can contribute back to life and lead a meaningful, fulfilling life and I learnt about this through the peer support … (S6-PW01-T1).

… being out of a job, out of work for a long time and feeling very hopeless about one’s future and experiencing high levels of mental distress oneself and thinking ‘God am I ever going to’, you know ‘what is the point of all this’, it’s just been a bit of a revelation I suppose that one can use it in a constructive way, that it hasn’t all been for nothing. … just knowing that one can understand probably more than many people … just trying or managing to make a small difference has been really helpful to me just for one’s own self-esteem really. (S4-PW02-T2).

Peer workers described a personal sense of reward from seeing the positive results of their peer support:

I think ultimately the real buzz of it is when you help people and then see the results and realise you have actually improved people’s quality of life either a little bit or a lot. (S4-PW05-T3).

I’ve found it rewarding. I’m really enjoying the job actually. I’m finding talking to people is really all I wanted to do and being such a people person I think this is the best job for me. I’ve learnt a lot from this role, it’s made a difference to my life. So it’s not only made a difference to the peer’s life but it has also made a difference to me. [S4-PW03-T2].

Proper remuneration for the role was symbolic of the value and recognition attached, by others, to the support that peer workers were providing:

… our peer coordinators explained to us that some of the Trusts have been just paying people on an as-and-when needed basis for this role whereas our is like a proper contracted permanent role. (S6-PW02-T2).

This has been my first paid job in probably 8 years I think … It’s been the most fantastic experience, I’ve absolutely loved it. (S1-PW05-T3).

However, that sense of value could be undermined where peer workers felt their role was misunderstood or not acknowledged by the clinicians they worked alongside:

I think it’s a mixed bag to be honest … I haven’t had any adverse things but sometimes I feel that they don’t understand the role and sometimes I think they can belittle it a bit. I don’t think sometimes people get how difficult it is to use your lived experience on a daily basis. [S2-PW06-T2].

Whenever I got a new service user I’d email their CPN or care coordinator, I’d send them a link even to the trial to give them more information about it and nobody apart from I think one person got back to me. So that’s been quite challenging not really having any communication or contact really with the mental health teams that are working with the service users that’s been a bit challenging and quite disheartening really as well in a way. [S6-PW03-T3].

Over time, peer workers hoped that the value of their work would be further recognised through ongoing employment (most of the peer workers in the study were on short term contracts for the duration of the research):

Well I just want to carry on … I’m glad to carry on as I’m doing at the moment and hopefully this will be a very long-term job to have. (S1-PW04-T2).

I’m hoping that once our contracts are up or not furthered or whatever more opportunities will come. (S3-PW05-T2).

For some, that sense of value or self-worth found in the role could be challenged when peer workers felt they were unable to offer enough support to people, or where the people they were supporting chose not to engage in peer support:

I think it’s disappointing when you have a limited time that you can spend with somebody. I’m surprised at how much I worry. I’m a worrier in any case but I really worry about my peers and how they are and I want to have more contact than just say an hour and a half or 2 h a week or however long it is. So, that for me is difficult. (S2-PW05-T3).

How to deal with non-engaging clients and you just, it is frustrating because you want to help them. You think the person would benefit … It feels frustrating that some of the clients I’ve worked with won’t say ‘right, yes I don’t want to take part, can I withdraw?’ And then that would free up a space for somebody else. (S5-PW02-T2).

One peer worker found it difficult to explain their role in social situations outside of the workplace where they did not feel comfortable disclosing their mental health difficulties, perhaps undermining the specific value of working in a peer support capacity:

… it does have a negative aspect in terms of outside of work when I have to explain my job to friends or strangers or whatever I kind of get that they don’t really understand what it is … their faces go really confused and … then having to explain it is another thing. I try and avoid saying using lived experience because then they’ll go down the route of ‘what do you mean, do you have a mental health?’ because I don’t want to expose myself and say that I have a mental condition or whatever … (S4-PW03-T1).

#### Feeling empowered

Many peer workers described a transformational impact of being a peer worker, acquiring a sense of purpose and providing opportunity for personal growth:

I think in the beginning there was just masses of self-doubt, am I doing this right, is that person thinking this of me, have I helped them, what if I’ve made them? Like loads of loads of anxieties, which I had to work quite hard to keep them small and not them grow. I think after I’d seen the full month through one or two times with different people, just when I think back now I’ve changed in so many ways I really have … (S1-PW03-T3).

It’s actually helping me to fit things in the rest of my life because it’s given a purpose, something that I want to do, something that I like doing and I know is going to create change in the future. So, it feels purposeful and I think that that is something that you learn through mental health as well, or mental illness, that to maintain happiness on a level there has to be a purposefulness there. (S2-PW05-T1).

… it was an empowering experience to be able to now be in a position to contribute and have a meaningful fulfilling role in life again by supporting others. (S6-PW01-T1).

Empowerment was found in being able to openly make use of experiences of mental health in the peer worker role:

I feel really empowered and hopeful, when I go into work I feel confident whereas in my last job those things weren’t there and really I felt, my last job didn’t know about my mental health and they had no idea about it and I felt like I … was putting that hat on where I had to be someone else. (S4-PW03-T1).

One peer worker reported, over time, how that personal development manifested first as aspiration for career development, and then to securing a new job:

The sky is the limit isn’t it. Anything is possible. I’d like to be able to achieve more, I mean I don’t know what the next level is in terms of becoming, once you are a peer support worker obviously maybe gain some more experience, maybe go on to a more senior role or managerial role. I’d like to do some training development for my own personal growth. And maybe go on to peer training. (S4-PW04-T1).

… the more I’ve continued on with this role my self-esteem and confidence has increased. So, I feel like I’m more how I used to be and I’m a lot more assured and assertive now than I was before, which brings me to the point that it’s given me the encouragement to be able to apply for other opportunities, which I have done, and I’m happy to say I’ve been successful … (S4-PW04-T3).

They indicated how the role had enabled them to acquire a wide range of skills and knowledge that might lead to opportunities beyond peer working:

… it’s taught me not just being a peer support work but also given me the opportunity to be able to train and deliver courses. It’s also given me the opportunity to learn other roles … it just has built up my knowledge base, skills and experience where now I’m in the hospital working within the mental health unit and I’m also still out in the community as well. (S4-PW04-T3).

The contribution of peer support training to an awakening sense of self-belief was also noted:

The training was giving me skills that I already had but that were lying dormant. The training supported that transformative process. So, the training in a way had a transformative impact on me in terms of empowering me and having more belief in myself. (S6-PW01-T1).

For one peer worker this transformational impact was articulated as a resilience against future adversity:

I started to get empowered by doing the stuff where I was learning from the peer support work and I thought, do you know what, they ain’t going to break me because I’m getting strong now and I’ve not learnt all these new skills and I’ve not learnt to get better just for my support network, which will be taken away, that they’re going to crush me because that ain’t going to happen now because I’ve changed. (S6-PW01-T1).

However, peer workers were clear that these positive impacts were only realised where balance in the demands of the role were achieved. One peer worker contrasted the current role with previous work that had been too demanding, while others described a process of establishing boundaries between work and personal life as they settled into the current role over time:

… so previously when I was at [name of service] they were very long days and it’s a crisis service so it’s extremely busy and you just don’t stop all day so I was tired. But this role, because it’s completely different, it’s good for me, it’s pushing me and I think it’s having a better impact on my wellbeing. (S5-PW03-T1).

I was so exhausted because I was getting so involved in doing all this work and doing this, that and the other and I wanted to do a good job and do extra little things, like I said to you I’m always working on my days off … I am slowly realising that, you know, that I’ve got to take some time for me … as stressful as it was at the time I’ve learnt a lot and I’ve learnt maybe what I should do next time … I think it’s a positive overall, I’m still enjoying it and I still enjoy seeing new peers so I think that says it all. (S2-PW07-T2).

#### Feeling connected

Feeling connected to others on a number of different levels was also a potential positive impact of working in the peer worker role. Peer workers described discovering a rewarding sense of connection through working with people in mental health services:

It was incredibly emotional because as soon as I walked on the ward … I was like, ‘I want to be back here on the ward with all these people’, and that was just in the back of my head. Obviously, I don’t want to go back in that way as a patient … I had to digest and reflect and see how I felt about that and that was a deciding factor for me because that was really important. Since I’ve been back it’s getting better and better, there’s no other way to describe it, it is actually the more I’m around, every time I come into work the more I’m around people, the times I go to the ward, it’s just like I’m on a complete high, happiness, I can’t believe this is so great. It’s just so positive. (S2-PW01-T1).

… it is a people role so it’s very communicative, it’s very social. So you can benefit from the reward of talking to someone else yourself. (S4-PW05-T3).

Peer workers described how they learnt from connecting with the people they were supporting, as well as from colleagues and professionals around them:

I like the interaction with the peers, I like the fact that I’m learning so much and it’s an ongoing learning process [and] experience … I like the people that I work with because I’m learning a lot from [Occupational Therapists], having interactions with the other clinicians etc. that work with the peers as well. (S4-PW04-T2).

I find it very interesting … to be able to meet people, fellow service users, that have struggled with mental health issues, it’s been pleasurable learning from them really because I learn from them as much as they learn from me. (S6-PW03-T3).

Another peer worker described how connecting with their colleagues as peers enabled them to better connect with their own experiences:

We really bonded so well because there was the ability to be open about our mental health and how it’s affected and to be able to listen to that. It’s quite traumatising to hear it from somebody else with your own story you … say for instance if I was speaking to a consultant or something I’d talk about my mental health like it’s happening to a different body. It was quite strange. And you become detached from it and this is about re-attachment to it. It opens your eyes because it puts you back in touch with what you experienced and you have those feelings right there raw, but it was such a safe place to do that. In way a comforting and in a way traumatising. (S2-PW05-T1).

However, this close connection, through peer support, with people who might share similar challenges to their own mental health was demanding for many peer workers:

It can be a bit of a challenge sometimes listening to people’s traumatic trust stories because of my personality disorder I feel quite emotional anyway. I don’t sit there crying in front of the service users but I can really feel their pain sometimes and that can be a bit difficult. (S6-PW03-T3).

One peer worker described how they needed to modify their work, with their manager, in order to address those demands:

I like connecting with people in life. So that’s why when I was feeling this intensity I said there was a burnout … and my manager said ‘what would help you’ and I said ‘I still need to continue to connect with people, I don’t want time off, but I just don’t want to connect as intensely as I was doing’. So, they needed someone to promote the project so me going out now and doing presentations was a win-win situation for the project and for me at this moment. So, it’s helped my mental health in those ways … I think I would like to do this maybe two or 3 days a week maximum. I think that’s probably the most healthy thing for me and do other things. (S2-PW04-T2).

Others found supervision or training on boundaries useful in helping them not to over-connect with the people they were supporting in their work:

To be honest I kind of switch myself off, I have to try and switch myself off. Supervision has taught me to do that … because I think sometimes you do tend to take your work home and you worry about things because you’ve built up such a rapport and a relationship with the peers it’s hard to detach yourself and walk away from it because you are concerned as any other person would be. But I suppose you are more emotionally attached as well because the nature of the role … if you’ve been through similar challenges then it’s even more difficult to let go of it as well. Our supervisor has been really good at enabling us to recognise those things and to reflect and say, ‘OK it’s alright to leave it on the table until next week’ … (S4-PW04-T3).

… the boundaries and relationships sessions that we did. I think boundaries has been vital really in this line of work … in terms of my boundaries, what I need to do to keep myself well and also be able to communicate and to, I think, limit sometimes the effect of something quite difficult that might be going on for that person and its effect for me maybe after work, so that switching off thing once I’ve dealt with that particular difficulty. (S4-PW01-T2).

Importantly, a supportive sense of connection was found through supervision (with the peer worker coordinator) and, mutually, with the peer worker team:

Supervision has been a really nice way to end the week because we all come together and we discuss ideas and issues and explore ways to make things better to improve our practice. I do enjoy working with my other peer workers as well, they’re really nice. We get on really well and I think having the people that you surround yourself with at work and a good work environment is really essential to your mental health … I would raise something that’s worrying me with my supervisor. So being able to share that is very helpful because then my supervisor will know what’s going on for me and might also say ‘OK I’m not going to give you a load of allocations when you’ve got some very difficult things going on for you’. So, part of that is also communicating to a supervisor or colleague what is going on is helpful because then you can get that support or support someone else. (S4-PW01-T2).

I think that was quite an apprehensive time for myself to know who I was going to be working with. But, yes, I get on really well with them, the people I did the training with I think we’ve built some good friendships there, some good trusting relationships. We feel quite comfortable to confide in one another which I think is key when you are doing a job like this. [Our supervisor] as well I think she’s very approachable, she was absolutely brilliant at the training and I think I’ve built some lasting relationships there which was, not surprising, but a nice addition to the training. (S5-PW01-T1).

The value of building relationships and developing a strong sense of team through the peer workers training was more widely recognised:

… we’re very, very supportive of one another. Inside and outside work. I think it’s probably because we all did the training together so we got to know each other over a period of time … It’s an ongoing relationship which has just become stronger as the time has gone by. So we give each other good advice, if there are any problems we share and air it out. If we can help each other in any way we tend to do that via email or a text or phone call. Yes, we share a lot of our resources … I guess we’re an extension of one another is the best way of describing it. [S4-PW04-T2].

Conversely, another peer worker noted a higher than expected level of independent working in the role, and having to manage working in isolation at times:

Well the level of responsibility and the level of freedom has surprised me. In a good way predominantly but it does have its moments. I think remote working is quite difficult or can be. It works both ways so it’s great to have the freedom and creativity and flexibility and whatever but on the other hand you can feel left with quite a lot of difficult feelings. The isolation is quite hard at times because you can feel like you are working a lot alone and having a lot of responsibility at times can feel too much. People say things which are challenging at times or slightly nerve wracking at times and you’ve got to manage that. (S4-PW02-T2).

Part of the transformative impact of peer support was a knock-on effect, enhancing connection to others, including family:

… it’s massively, massively improved my wellbeing. It’s been transforming. Everybody has seen a difference … like my family, I’ve been able to talk to my mother. There was a time when I was not able really to talk to my mother and I don’t think she’s long for this world, but the thing is she is so happy, and she’s seen the changes in me and now we’re able to stay in the same room and have a conversation without it turning into a row or an argument … I’ve been able to make contact with people who ... didn’t want to know me before. … getting this role has transformed myself in terms of how I see myself and how others see me. (S6-PW01-T1).

… it definitely has helped me in my personal life because it’s actually changed how I talk to my children and specifically my 14-year-old, my first child. I hadn’t been listening, I had just been in mum role and just giving orders and stuff. Our relationship has changed since I’ve been [working as a peer worker] and he actually has started telling me more things and opening up and telling me things that I had no idea was even going on. With my mum as well that’s changed, with my best friend. (S2-PW01-T1).

Finally, while the sense of connection within the peer worker team was experienced as good, a number of peer workers reported a relative lack of connection, or tension with the clinical teams who were providing care with the people they were supporting, impacting on the quality of support they were able to provide:

… it’s been a bit blurred about what our role is as peer support workers [when] there hasn’t been a care coordinator or other support workers involved with a particular peer that you are working with. I think sometimes you are a little bit forced into helping with things like accommodation … and that’s not your role. So that can sometimes be a bit difficult if they are not getting the contact with particular other people as often as they want. (S4-PW01-T2).

… what you find is sometimes having that engagement, that rapport with them [clinical teams] is actually very supportive of one another and sometimes that’s what you need to be doing because it’s not about working against anyone, it’s about working together and it’s not about stepping on anyone’s shoes either whereas you’re coming in and you’re taking over and perhaps your rapport is better than the clinicians and other key workers or care coordinators or support workers that are involved. … I do believe that has happened and it’s not meant to be like that because we’re all supposed to be reading off the same page. (S4-PW04-T2).

### Synthesis

Quantitative and qualitative data were synthesised as described above, resulting in five main synthesising propositions. These are presented in Table 5 and used to inform the discussion that follows.

**Table 5:** Synthesis of quantitative and qualitative analyses

Proposition	Quantitative analysis	Qualitative analysis
1. Peer worker wellbeing is generally good and remains so over time in the role	Peer worker wellbeing scores post-training were similar to those of the general public and remained so over 12 months Peer worker burnout scores post-training higher than comparable healthcare professionals and remain so over 12 months Days absent from work for peer workers were similar to wider UK mental health workforce Mental health and wellbeing service use for peer workers was generally low and changed little over 12 months	Feeling valued and rewarded, and a transformative sense of empowerment, purpose and self-worth - gained through acquisition of skills, training and experience of supporting others and being able to make use of own experiences of mental health - were important impacts of working in the peer worker role The peer worker role could be emotionally demanding, or the sense of value and self-worth undermined where peer workers felt unable to offer enough support or the people they worked with discontinued peer support
2. Job satisfaction for peer workers and experiences of multi-disciplinary teamworking are generally good and remain so over time in the role	Peer worker job satisfaction and multi-disciplinary team working scores post-training were similar to comparable groups of healthcare professionals and remained so over 12 months	Proper remuneration in the peer worker role was symbolic of value and recognition although this could be undermined where clinicians did not understand or acknowledge the peer worker role
3. Peer worker wellbeing and some aspects of job satisfaction can drop, and some feelings of burnout increase over the first few months in post, but then stabilise as peer workers adjust to, and are properly supported in the role	There was a significant drop in wellbeing, personal satisfaction and satisfaction with workload, and an increase in the depersonalisation burnout subscale at four months (only the latter was maintained at 12 months, and all scores remained comparable to or better than norms)	Peer workers could find the emotional and practical demands of the role difficult to manage in the first few months in post, but could find balance in the role through adapting and adjusting their approach and workload with the support of their supervisor
4. Peer worker satisfaction with job prospects and training can drop over time where job certainty and career development opportunities are unclear	The was a significant drop in peer workers’ satisfaction with job prospects scores at four and 12 months in the post, and in satisfaction with training at 12 months	Peer workers were initially extremely optimistic about ongoing job prospects – whether that involved more peer support or moving into other work - while over time peer workers became less optimistic, expressing hope that their contracts would be continued in the face of uncertainty Initial training was very well received. Peer workers expressed interest in further training and career development opportunities
5. An enhanced sense of connection to self and others, within and beyond work, is an important impact of working in the peer worker role (Note: good communication with clinical colleagues is needed as part of that sense of feeling well connected in the peer worker role)	De-personalisation subscale scores on the burnout measure are more than one SD lower than a comparable group of healthcare professionals While non-significant, there was a reasonably large reduction the communication subscale of the multi-disciplinary team measure at four and 12-months in post	Connecting with people they were supporting and the peer workers they worked alongside was a learning experience for peer workers, enabling peer workers to better connect with their own mental health The close connection experienced through offering peer support could be demanding, although a strong sense of connection with the peer worker team, developed while training together and further experienced through supervision, was supportive There could be a positive knock-on effect on improved connection with family and friends There was sometimes a need to improve communication and a sense of connection with the clinical teams that peer workers worked alongside

## Discussion

This study aimed both to measure the impact of working as a mental health peer worker on wellbeing and employment-related outcomes, and to explore the experience of impact through in-depth interviews with peer workers. We note that the focus of our enquiry was concerned more with understanding and evaluating the demands, rewards and sustainability of the peer worker role, rather than asking if being a peer worker has a positive impact on the mental health of individuals taking on the role. Thus, while we observed a small downward trend in many of the outcomes we measured, changes were largely non-significant over the course of a year and scores remained as good or better than those for comparable populations (and qualitative experiences were often positive). We consider our findings in that context.

We observed levels of wellbeing among peer workers comparable to the general population when they came into the role and remaining so over the course of a year. The construct of wellbeing as measured here [36], comprised components focused on subjective experience of happiness and life satisfaction, and on psychological functioning and self-realisation [51]. Our analysis of qualitative interview data suggested that the positive impacts of working in the peer worker role – of feeling valued, empowered and connected – broadly reflected that construct. On the one hand, peer workers experienced a ‘buzz’ and ‘excitement’, as well as strong sense of achievement in the role, and on the other a transformative sense of personal growth alongside increased confidence and self-esteem. Peer workers felt empowered by the role and that it had brought a new meaning and purpose to their lives, aspects of wellness associated with peer support work found in other research [8, 9, 13]. While measuring somewhat different constructs, this positive sense of wellbeing was reflected in scores in the ‘personal achievement’ burnout subscale [24] that were higher than normative data, and high levels of job satisfaction [37], also at starting work and for the most part remaining so over the next year. Elsewhere, high levels of satisfaction with being a peer worker have been associated with pay and working conditions alongside enjoyment of the work [22], with peer workers in our study responding positively to being paid on substantive, rather than casual contracts. On a more functional level, peer workers did not access acute or emergency mental healthcare during our study, while sickness absence was comparable with that across mental health staff in the NHS in England [29]. As such, peer workers were well when they came into role and largely remained that way, challenging views voiced by some mental healthcare professionals that peer workers might inevitably become unwell when faced by the stresses of peer work [6]. Research has also indicated the importance of taking care in recruiting people who are ‘ready’ to take on the role [11].

The peer worker benchmarking exercise in England estimated sickness absences from work for peer workers in 2019 at 22% [29], considerably higher than levels of absence across the mental health workforce. Noting the possibility that peer workers were largely compared, in that exercise, to established professionals in more secure, better supported posts, this was not the case in our study, reflecting similar findings from Australia [28]. This variation in absence rates suggests that other factors, perhaps relating to working conditions [52] or the organisational support and training provided for peer workers [53, 54], might be impacting absence and turnover. Qualitative data in our study strongly suggested that supervision and support from the peer worker team were crucial in managing the demands of the role, with above norm scores also reported in almost all subscales of the multidisciplinary team measure [38]. Some peer workers did report finding the work both emotionally draining and practically demanding, at least in the period immediately following starting work, reflected in a drop in wellbeing scores, and some burnout and job satisfaction subscale scores at 4 months. We note that this drop in scores was not, on the whole, maintained at 1 year, and a US longitudinal study with peer workers in veterans’ mental health services similarly observed an initial increase in burnout (at 6 months) that was not maintained at 1 year [27]. Data in our study suggested that initial high scores, perhaps buoyed by optimism at taking on a new role, were tempered somewhat as the realities of the job sank in, but that as peer workers became further accustomed to the role there was no sustained decline at 12 months. Qualitative data supported that explanation, with peer workers reporting at 4 months having had to adjust to the responsibility they had taken on and needing to better manage their workload, finding ways to cope with the intensity of connecting with people as peers with the support of their supervisor. A recent systematic review of literature exploring factors shaping the implementation of peer support in mental health services identified the importance of appropriate supervision as highlighted across multiple studies [10]. The implication of these findings for practice is that organisations employing peer workers need to be aware of this potentially challenging time for peer workers in the first few months after coming in to post, ensuring that support and supervision, both practical and emotional, is in place to enable peer workers to successfully adjust to the demands of the role [18]. Nonetheless there were two areas where satisfaction with the role did drop – employment prospects and access to training – with the optimism around future employment (either within or outside of peer support) that peer workers initially expressed starting to wane as fixed term contracts came towards to an end and ongoing job security became less certain for some. The wider peer support literature has indicated the importance of continued, on-the-job career development support, including advancement and promotion in the role [19].

Our qualitative data indicated clearly how peer workers experienced an enhanced sense of connection through peer working, derived to a large extent through a supportive experience of being part of a peer worker team. In contrast, earlier research had noted how a lack of opportunities to network with other peer workers hindered the successful implementation of peer support in mental health services [55]. Peer workers in our study also spoke about a greater sense of connection with self, realised in part through interacting with, and learning from the people they were supporting. This reflects the idea of reciprocal learning that has been identified as core to the concept of peer support [56] and embraced as a core value underpinning peer support as it was implemented in this study [33]. Scores on the depersonalisation subscale of the burnout measure [24] were considerably lower (less burned out) for peer workers in our study compared to a normative sample of mental healthcare workers, again reflecting this experience of peer support as enabling self-growth through reciprocal connection. In contrast, both our qualitative data and scores on the communication subscale of the interdisciplinary team measure [38] suggest that connection with the clinical team was not as strong. While tension with clinical team members has been identified as inimical to successful implementation of peer support [16, 18, 57], research has shown that peer workers have felt more integrated into teams as clinicians began to appreciate the value of peer support and attitudes changed [55, 58]. It is important to note that in the ENRICH peer support approach, peer workers were managed within their own peer support team, working across inpatient and community mental health services as necessary, rather than being embedded as part of the complement of the ward or community multidisciplinary team. Our findings suggest that this organisational arrangement contributed to a strong, positive, supportive sense of (peer worker) team, potentially circumventing many of the challenges associated with integrating into the multi-disciplinary team that have been identified as an additional source of stress for peer workers [16, 17]. However, the drawback of this arrangement was that peer workers might not have had the opportunity to build that shared understanding of their role with clinicians [13], in their view, potentially hampering their ability to provide the best possible support. While other research has identified that peer workers make a distinctive and highly valued contribution to the multi-disciplinary mental health team [59], more research needs to be done in order to establish an organisational model that optimises the potential for peer support either alongside or within the clinical team.

A strength of this study was its longitudinal nature and the use of both quantitative measures and qualitative data exploring peer workers experiences of working in the role, a first study in the field of this design. In particular, having an interim time point allowed us to identify challenges at a point when peer workers had started providing face-to-face peer support but did not yet feel established in the role. Qualitative data allowed us to make further sense of how changes in outcome might be associated with working conditions and the support peer workers received, and also extended the scope of the enquiry, enabling us to explore, for example, how of a sense of connection with self and others, through peer working, affected positive impact of the role. However, our sample was small and outcomes data were often incomplete at follow up, limiting the power of our analyses and our ability to explore possible associations between outcomes. In addition, the standardised outcome measures we used, although demonstrating good face validity, have not, to the best of our knowledge, been formally validated for use with peer workers. Similarly, our data on peer worker absences from work was not collected in a similar way to the mental health workforce data and lacked information on reasons for absence and so on. Prospective research would make more reliable comparisons. A future study might also seek to assess how impacts identified in the qualitative study, of feeling valued, empowered and connected, are associated with the quality of support that peer workers are offered.

In conclusion, our study finds that peer workers largely stay well and experience a positive sense of self and growth in their work, including in demanding roles based in acute mental health services, where they are well supported and valued in that capacity. We note that particular attention needs to be taken to providing appropriate support for newly employed peer workers as they accustom themselves to the emotional and occupational demands of the role (including supervision focused on these early challenges). As peer workers continue in post, in-work training that builds on a basic peer support training, and efforts to improve job insecurity are important so that the positivity peer workers bring to their work is not undermined. Finally, we note that basing peer workers within a dedicated peer support team is experienced as highly supportive by peer workers but might hamper the optimal provision of peer support where connection to clinical teams is not also supported.

## Data Availability

The datasets used and analysed during the current study are available from the corresponding author on reasonable request.
